# Early BCR Events and Antigen Capture, Processing, and Loading on MHC Class II on B Cells

**DOI:** 10.3389/fimmu.2014.00092

**Published:** 2014-03-10

**Authors:** Ana M. Avalos, Hidde L. Ploegh

**Affiliations:** ^1^Whitehead Institute for Biomedical Research, Cambridge, MA, USA

**Keywords:** B cell receptor, antigen valency, antigen recognition by BCR, antigen presentation by B cells, B cells as APC

## Abstract

B cells are efficient antigen-presenting cells (APCs), relying on antigen uptake through the B cell receptor (BCR). The mechanism of antigen recognition remains a topic of debate; while the prevalent view holds that antigens need to be multivalent for BCR activation, monovalent antigens can also initiate B cell responses. In this review, we describe the steps required for antigen uptake, processing, and loading of peptides onto MHC Class II compartments in B cells for efficient presentation to CD4 T cells, with a special focus in the initial steps of BCR recognition of antigen.

## B Cells as Antigen-Presenting Cells

Professional antigen-presenting cells (APCs) take up antigen through phagocytosis, fluid-phase pinocytosis, or receptor-mediated endocytosis for processing, loading of the digestion products onto MHC Class II (MHC II) and presentation to CD4 T cells. The ability of B cells to present antigen to CD4 T cells was recognized over three decades ago, but they were deemed less efficient than dendritic cells or macrophages due to their comparatively lower capacity to take up antigen non-specifically. In contrast, antigen recognition through the BCR is a far better (up to 10,000-fold) means to present antigen to CD4 T cells compared to non-specific antigen uptake (1). BCR ligation by antigen leads not only to antigen capture and antigen delivery to MHCII compartments, but also initiates signaling and *de novo* gene expression. This, in turn, influences the activation state of the B cells and their ability to engage CD4 T cell help. The nature of the antigen and strength of interaction (affinity) for BCR are thus essential features that determine whether processed antigen can be efficiently presented to CD4 T cells. Here, we review the sequential steps required for BCR recognition of antigen, internalization, processing, and loading onto MHC II, with special emphasis on the early events of BCR–antigen interaction required for efficient B cell activation.

## Antigen Capture by B Cells

Antigen recognition by naïve circulating B cells occurs in follicles present in peripheral lymphoid organs (lymph nodes and spleen), which receive continuous inputs by means of lymphatic fluid originating in peripheral tissues and delivered to lymph nodes through the afferent vessels (lymph node) or via blood, which carries lymphocytes and antigen through the trabecular artery (spleen) (2). Small, soluble antigens (<70 kDa) quickly (within 2 h) reach follicular B cells through conduits that penetrate the follicle (3); larger antigens such as viruses or immune complexes (ICs) are retained within the subcapsular sinus by macrophages and follicular dendritic cells (FDCs) that capture and present antigen to B cells by tethering via complement (CD21 and CD35) or Fcγ receptors (4–6). Antigen presented on the cell surface is recognized by the BCR through the formation of a macromolecular cluster of defined composition, the immunological synapse (IS) (7). At the IS, B cells recruit and secrete MHC II-positive lysosomes in a microtubule-dependent polarized fashion. The small GTPase Cdc42 is required for lysosome exocytosis, which results in acidification of the IS extracellular space and may facilitate removal of antigen from the presenting cell and/or proteolysis (8). The cellular contacts between APCs and B cells last 20–30 min (9), a timeframe that allows sustained BCR signaling and antigen capture. Antigen is captured by BCR in a process that also brings in membrane components by physical extraction from the presenting cells (7). The efficiency of this process is the result of the affinity of BCR–antigen interaction: stronger binders will be more likely to be “pulled” through mechanical forces by the B cell (10). Higher affinity for antigen also translates into enhanced CD4 T cell activation (11). Upon antigen internalization, cognate B cells migrate to the boundary of the B and T cell zones, aided by recognition of CCL19 and CCL21 [secreted by stromal T cells (12)] through the CC chemokine receptor 7 (CCR7) for recruitment of T cell help. Successful engagement of CD4 T cells can lead to formation of germinal centers (GCs), where affinity maturation and isotype switching occur. In the process of migration, B cells that have internalized antigen need to digest it and load the resulting peptides onto MHC II molecules for presentation to CD4 T cells. How does BCR recognition of antigen permit antigen uptake, processing, and presentation on MHC II?

## Early Events of BCR Recognition of Antigen

The BCR discriminates tonic signals delivered to B cells in their resting state, required for survival, from activating signals that lead to differentiation and antibody production. It is generally accepted that antigen binding leads to BCR clustering and internalization of antigen; however, the steps required to achieve such clustering, the antigen valency, and even whether clustering is required at all for activation remains controversial. Total internal reflection fluorescence microscopy suggests that most BCRs are monomeric on the cell surface and aggregate upon ligand binding (13) in a manner dependent on the Cμ4 domain of BCR (14). However, studies on insect cells reconstituted with the BCR complex indicate that BCRs are oligomeric and autoinhibited complexes at rest; upon ligand binding they undergo a conformational change to a monomeric “active” state (15). Indeed, IgD and IgM were found to form clusters on the surface of resting cells, though their nanoscale organization remained mostly unaltered upon antigen binding unless the BCR was heavily crosslinked using anti-Ig complexes (16). Thus, the BCR may be distributed on the cell surface in equilibrium between clusters of different size and number, versus monomers, and aggregation may not be a strict requirement for activation. Such distribution may allow tonic BCR signals necessary for survival.

Diffusion of the BCR in resting cells is constrained by the actin cytoskeleton: areas rich in actin and the ezrin–radixin–moesin (ERM) family of proteins, which are proteins that link plasma membrane components to the actin cytoskeleton, are associated with slow-diffusing BCRs. Alteration of the actin cytoskeleton by exposure of B cells to depolymerizing agents such as Latrunculin A or Cytochalasin D leads to BCR signaling in the absence of antigen, and increase the rate of diffusion of the BCR (17). The tetraspanin CD81 is required for BCR signaling upon actin disruption and antigen binding (16). Actin plays an essential role in antigen capture; upon antigen encounter, B cells undergo a spreading response, presumably to help capture more antigen at those locations where actin depolymerizes and BCRs diffuse more rapidly. This response is followed by a contraction response, clustering of BCRs and antigen, and actin polymerization. Both spreading and contraction responses depend on signaling-competent BCRs (18). Thus, the actin cytoskeleton helps control BCR dynamics, both at rest and upon activation.

**Figure 1:**
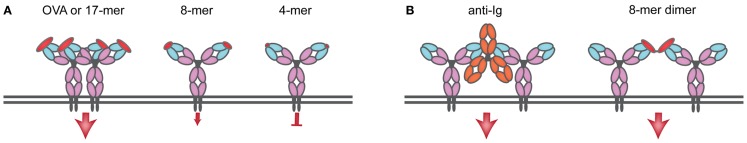
**Monomeric antigen can induce BCR cross-linking and activation as long as a signaling threshold is attained**. **(A)** OB1 B cells bear a BCR specific for ovalbumin (OVA), and activation and cross-linking is produced upon interaction with a 17-mer peptide containing the OB1 epitope and an essential FGD sequence. As the size of the peptide including the FGD sequence decreases, so does the ability to induce signal: an 8-mer peptide fails to produce above-threshold signals and it is suboptimal; a 4-mer peptide (GFGD) fails to induce any signal. **(B)** Reagents directed to the constant region of IgG (such as anti-IgG or anti-kappa) cross-link the BCR by bringing BCRs in close apposition, a similar effect is produced upon chemical conjugation of 8-mer to produce dimers that can now induce significant (above-threshold) BCR signaling.

The nature of antigen is critical in determining the different steps of B cell activation. The current view that antigen needs to be multivalent in order to crosslink the BCR (19) has been challenged by a few studies that show that monovalent engagement of the BCR can elicit B cell activation (20–22). Indeed, antigens that are unable to simply crosslink the BCR have the ability of activating B cells (23). One of the few reports that have correlated the valency of antigen with outcome of B cell activation used the Hen Egg Lysozyme (HEL) BCR transgenic model (24). HEL crosslinked chemically and then resolved by size exclusion chromatography was used to obtain dimers, trimers, and tetramers, which were then compared with HEL monomers for their ability to activate B cells. While HEL monomers and multimers elicited comparable early BCR signaling events, monomers were less efficient at presenting antigen to cognate CD4 T cells (20). Another study used the hapten nitro-iodophenol (NIP)-specific B1–8 mouse and 8–12 residue peptides bearing different numbers of NIP linked to the ε-amine of lysine residue incorporated into the peptide. Peptides bearing low numbers of NIP (two and three) molecules could activate B cells as read out by tyrosine phosphorylation and Ca^2+^ flux, but did so inefficiently, while monomers failed to induce any response. Surprisingly, dimers could activate equally well, regardless of whether NIP-molecules were placed on adjacent lysines or whether separated by 24 amino acids, a finding difficult to reconcile with the expected molecular distances between variable regions in the same BCR or achievable upon clustering (21). The question of antigen valency has been difficult to address because the existing transgenic BCR models are specific for molecules whose valency cannot be carefully controlled *in vitro*: DNA is a multivalent antigen with a repetitive structure, and it is technically challenging to demonstrate the absence of protein aggregates in solution. While free hapten can bind to an antibody of the appropriate structure, free hapten does not activate B cells; for immunization, haptens require association to carrier molecules where the position of the hapten on the carrier is not known and in any case is highly variable (24–26). In an attempt to answer the question of valency required for activation of antigen specific B cells, we resorted to transnuclear mice specific for ovalbumin (OVA) for which the peptide epitope was mapped to the 10-amino acid peptide DKLPG**FGD**SI in which the FGD sequence is essential for recognition (27). A 17-mer peptide centered on the FGD epitope promoted early BCR responses as strongly as did OVA (Figure 1A). Shorter peptides still bearing the FGD epitope progressively lost the ability to activate B cells, with an 8-mer version yielding suboptimal signals and a 4-mer version being inert (Figure 1A). Monovalent peptide promoted expression of CD86 activation marker, but less so than that evoked by the natural ligand, OVA. In unstimulated OB1 B cells, most IgG BCR remained monomeric, and 17-mer-peptide incubation lead to clustering of the BCR, internalization and localization to MHC II compartments (22). Thus, monovalent antigens can trigger BCR responses but only when early signals may exceed a threshold required to elicit BCR optimal downstream signaling and expression of activation markers, leading to BCR clustering (Figure 1A). Less efficient monovalent antigens can bind and trigger proximal BCR events but the signal may not be of sufficient duration or strength to elicit activation marker expression. Agents capable of crosslinking the BCR and commonly used to activate polyclonal B cells [such as the widely used F(ab)′2 anti-IgM] may simply “bypass” the specificity threshold barrier by enforcing close apposition of BCRs (Figure 1B). Indeed, by chemically conjugating 8-mer (suboptimal) peptides, we were able to create optimal responses (Figure 1B). Thus, upon BCR ligation two types of signals may emerge that depend on the valency of antigen and the physical disposition (clustered or free) of the BCR; monovalent antigen may trigger a signal that needs to overcome a threshold to start the activation process and clustering, and polyvalent antigen may ligate already clustered BCRs and/or bring BCRs closely together. Monovalent membrane-bound antigens are more efficient at triggering BCR signals and at antigen presentation to CD4 T cells when compared to their soluble counterparts, possibly due to a high local concentration of antigen in the two-dimensional structure of the IS (7). Perhaps monovalent BCR engagement may be more prevalent for membrane-bound antigens though soluble monovalent antigens can mediate activation as well.

## BCR Signaling and Antigen Internalization

Upon antigen recognition by the BCR, the kinase Lyn phosphorylates immunoreceptor tyrosine activating motifs (ITAMs) in the non-covalently associated Igα and Igβ heterodimers within seconds. This leads to Syk kinase and PLCγ2 recruitment and phosphorylation, Ca^2+^ release from endoplasmic reticulum and MAPK activation. The stimulation of these many signaling pathways triggers the transcription factors NFAT, NFκB, and Fos, Jun, and Ets [BCR signaling pathways reviewed in Ref. (28, 29)]. Signaling and internalization of antigen are interdependent events. Internalization through clathrin-coated pits is dependent on tyrosine phosphorylation of the heavy chain of clathrin by Src-family kinases localized in lipid rafts (30). As antigen is brought in, signaling continues as phosphorylated early kinases (Lyn and Syk) remain associated to BCR until its trafficking to lysosomes; later phosphorylated kinases such as Jnk, p-38, and Erk first associated at the plasma membrane-early endosome interphase, continued to accumulate in multivesicular bodies (MVB, 200–350 nm diameter, the sites of antigen processing and loading onto MHC II). Treatment with the dynamin inhibitor dynasore prevents clathrin-mediated endocytosis and leads to hyperphosphorylation of Lyn, Syk, Jnk, p-38, and Erk kinases, and hypophosporylation of Akt. As a result, gene expression was dysregulated suggesting that endocytosis is involved not only in antigen internalization but is also required for signaling (31) presumably from endocytic compartments themselves.

## Antigen Processing and Loading to MHCII

**Figure 2:**
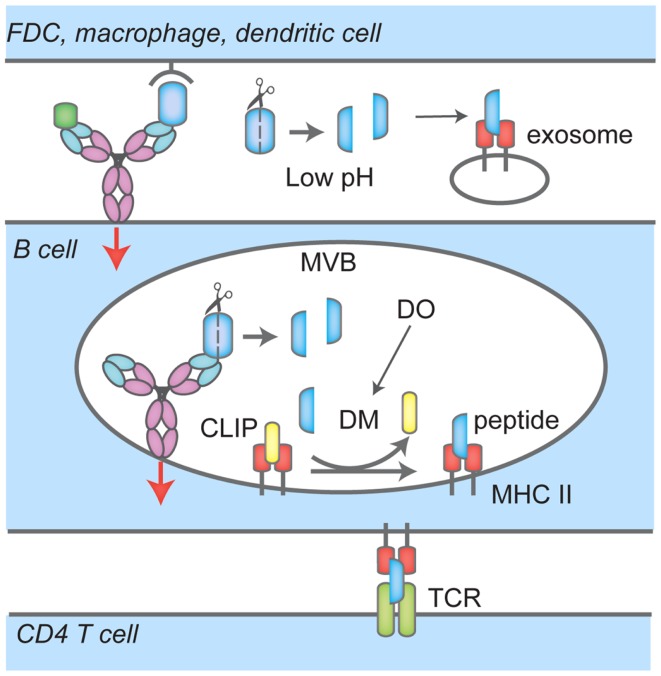
**Events that lead to antigen uptake by the BCR, loading onto MHC Class II and presentation to CD4 T cells**. BCR interacts with soluble antigen, or antigen presented by follicular dendritic cells (FDC), macrophages or dendritic cells at the immunological synapse (IS), leading to signaling events (red arrow), antigen-BCR complex uptake and translocation to multi-vesicular bodies (MVB). There, proteases cleave antigen into peptides that are loaded onto MHC Class II; H2/HLA-DM (DM) mediates the removal of the class II-associated peptide (CLIP) which prevents MHC Class II premature loading. H2/HLA-DO (DO) regulates DM. In these compartments, BCR signaling continues (red arrow). B cells also recruit and secrete MHC II-lysosomes to the IS space in a polarized fashion, lowering the pH and facilitating removal of antigen or proteolysis. Peptide-MHC II complexes are then transported to the cell surface for presentation to CD4 T cells.

In the ER, newly synthesized MHC II αβ dimers associate with the invariant chain (Ii), a chaperone that delivers MHC II to the endocytic pathway. There, proteases such as Cathepsin S and L stepwise cleave Ii to finally yield the class-II-associated invariant chain peptide, CLIP, which remains associated with the peptide-binding groove to prevent premature loading with peptides. MHC II has been shown to localize to unique MIIC compartments, distinct from endosomes or lysosomes (32, 33). In B cells, it has been shown that upon BCR ligation by F(ab)′2 specific for Ig, MHC II dimers are redistributed to LAMP1 positive MVBs (34). The distribution of Class II MHC products over MVB, lysosomes, and other endosomal structures in response to BCR engagement remains a matter of debate. Upon BCR ligation, BCR–antigen complexes are brought into MHC II-rich compartments through the endocytic pathway, and the heavy chain of BCR (35) as well as Igα and Igβ (36) are ubiquitylated. Igβ is ubiquitylated at the plasma membrane by the E3 ligase Itch, a process dispensable for BCR–antigen internalization but required for sorting to compartments bearing the lysosomal marker LAMP1+ and antigen presentation (36). BCR ubiquitylation also depends on Syk signaling (37). In MHC II-rich compartments, H2/HLA-DM (DM), removes CLIP peptide from MHC II to allow loading with antigenic peptides (Figure 2). In B cells, these processes are reversible and the B cell phenotype, as assessed by surface markers, returns to that of unstimulated cells after 24 h (34). DM not only removes CLIP to permit binding of antigenic peptides to the peptide cleft of MHC II, but also plays a role in selecting strong binders from weak binders (38). B cells express the chaperone HLA-DO (DO), a DM inhibitor (39) except for when MHC II are localized to acidic compartments; this mode of inhibition ensures that only peptides that are a result of proteolytic degradation of antigens internalized through the BCR are loaded onto MHC II (38) (Figure 2). As expected, DO expression is reduced in germinal center B cells, which are competent APCs (40).

The actin cytoskeleton plays a role in antigen presentation by B cells; its regulation is shared with components of the BCR pathway such as Syk, Btk, and Vav (41). The actin motor myosin II protein also regulates MHC II trafficking and antigen presentation, through Ii-dependent interaction with Class II MHC molecules (42). As mentioned before, B cells polarize the microtubule-organizing center and Class II MHC compartments toward the point of first contact with antigen upon antigen binding, a step required for normal B cell function. B cells maintain a polarized antigen distribution upon division, which leads to unequal partitioning of captured antigen over daughter cells: the mother cell contains a full antigenic load while daughter cells are devoid of antigen. This asymmetrical distribution of antigen leads to unequal antigen presentation capabilities; antigen content correlates with the ability to present antigen to CD4 T cells. However, symmetrical division and antigen segregation do occur and the diminishing presentation capabilities of daughter cells may also derive from progressive dilution of antigen in cells that divided symmetrically (43). Thus, the actin cytoskeleton plays essential roles not only in the initiation of the processes required for efficient antigen uptake but also in loading onto MHC II for presentation to CD4 T cells.

## Concluding Remarks

B cells are programed to present antigen to T cells primarily after BCR-mediated internalization of the antigen. The process of antigen recognition through the BCR not only triggers drastic changes in B cell gene expression profiling, but also affects endocytic trafficking and surface molecule expression. These steps require antigens that are sufficiently strong to exceed the threshold required for activation. When this criterion is met, BCR clusters, B cells expand in size, with concomitant enhanced diffusion rates of the BCR to capture more antigens. B cells then contract, internalize antigen, proteolyze it and present the resulting peptides on Class II MHC products. How exactly these processes are coordinated remains to be uncovered: is there a minimum BCR signaling level required to internalize antigen, or regardless of the signal strength will any antigen bearing the epitope be internalized? Is BCR crosslinking required for triggering gene expression, internalization, and antigen presentation, or just for a few of these processes? The valency of antigen and affinity are essential determinants to fully activate these processes, but which aspects of signaling and antigen presentation are directly affected remain to be established. B cell survival requires tonic signaling via the BCR, and activation triggers must therefore be finely tuned to prevent activation by non-optimal BCR ligands and autoantigens. A better understanding of how BCR recognition of antigen is tuned to control downstream processes and outcomes of B cell activation will require additional BCR animal models with homogeneous B cell populations; these models will enhance our knowledge for the rational design of therapies aimed to boost B cell responses or control B cell malignancies and autoimmunity.

## Conflict of Interest Statement

The authors declare that the research was conducted in the absence of any commercial or financial relationships that could be construed as a potential conflict of interest.
